# Genomic characterization of *Vibrio cholerae* isolated from clinical and environmental sources during the 2022–2023 cholera outbreak in Kenya

**DOI:** 10.3389/fmicb.2025.1603736

**Published:** 2025-07-07

**Authors:** Lydia M. Mageto, Gabriel Oluga Aboge, Zelalem H. Mekuria, Peter Gathura, John Juma, Michael Mugo, Collins Kipkorir Kebenei, Diana Imoli, Beatrice Atieno Ongadi, Kelvin Kering, Cecilia Kathure Mbae, Samuel Kariuki

**Affiliations:** ^1^Department of Public Health, Pharmacology and Toxicology, Faculty of Veterinary Medicine, University of Nairobi, Nairobi, Kenya; ^2^Washington State University, Global Health Kenya, Nairobi, Kenya; ^3^Department of Veterinary Preventive Medicine, College of Veterinary Medicine, The Ohio State University, Columbus, OH, United States; ^4^International Livestock Research Institute (ILRI), Nairobi, Kenya; ^5^Centre for Microbiology Research, Kenya Medical Research Institute, Nairobi, Kenya

**Keywords:** cholera, antimicrobial resistance, whole genome sequencing, phylogenetic analysis, virulence

## Abstract

**Background:**

Cholera remains a public health challenge in Kenya. To better understand its dynamics, we analyzed *Vibrio cholerae* genomes from clinical and environmental samples collected during the 2022–2023 outbreak. These strains were compared with historical genomes from Kenya, Uganda, Tanzania, and Haiti to inform strategies for cholera prevention, control, and elimination in Kenya.

**Methods:**

Clinical (stool) and environmental (wastewater, drinking water, and household effluent) samples were collected from Nairobi county. Samples were analyzed for *V. cholerae* using culture and real time PCR. The environmental (*n* = 17) and clinical (*n* = 70) isolates were then subjected to phenotypic antimicrobial susceptibility testing using the Kirby-Bauer disk diffusion method. Whole genome sequencing was employed to characterize the genome, detect antimicrobial resistance genes, virulence factors, and mobile genetic elements. Phylogenetic analysis was performed to assess the genetic relationship and diversity of isolates from 2022 to 2023 outbreak, comparing them with isolates from historical outbreaks.

**Results:**

Clinical isolates carried key virulence genes (*ctxA, ctxB7, zot*, and *hlyA*) and were 100% resistant to multiple antibiotics, including ampicillin, cefotaxime, ceftriaxone, and cefpodoxime, but remained susceptible to gentamicin and chloramphenicol. In contrast, environmental isolates lacked *ctxB* gene but harbored *toxR, als*, and *hlyA*, showing variable antibiotic resistance (59% to ampicillin, 41% to trimethoprim-sulfamethoxazole, and 47% to nalidixic acid). All clinical isolates from 2022 to 2023 outbreak harbored IncA/C2 plasmids and several antimicrobial resistance genes including *bla*_*PER–*7_. Phylogenetic analysis revealed high genetic diversity in environmental strains, clustering outside the 7th pandemic El Tor lineage, while clinical isolates were highly clonal. Genomes from 2022 to 2023 outbreak were closely related to Kenyan cholera outbreak genomes from 2016 (15 single nucleotide polymorphisms, T13 lineage).

**Conclusion:**

The 2022–2023 outbreak likely resulted from re-emergence of previously circulating strains rather than a new introduction. While the role of environmental reservoirs as a source of human infection remains unclear in our study, environmental isolates possess virulent and antimicrobial resistance genes that may spread via horizontal gene transfer. This highlights the need for continuous genomic surveillance to monitor *V. cholerae* evolution, track transmission patterns, and mitigate the spread of antimicrobial resistance.

## 1 Introduction

*Vibrio cholerae*, the causative agent of cholera is transmitted through consumption of contaminated water or food (Harris et al., 2012). Globally, the burden of cholera in endemic countries is estimated at 1.3–4 million infections with 143,000 mortalities annually (Ali et al., 2015). Out of the 209 different serogroups of *V. cholerae*, only serogroups O1 and O139 are known to potentially cause epidemics (Chun et al., 2009). Non-O1/non-O139 (NOVC) serogroups are considered environmental strains and part of the normal flora in aquatic ecosystems. However, certain NOVC strains can cause cholera outbreaks characterized by mild diarrhea as reported in a number of countries (Dalsgaard et al., 1999; Chatterjee et al., 2009; Onifade et al., 2011). *V. cholerae* O1 is categorized into El Tor and classical biotypes based on their phenotypic traits (Tappero and Tauxe, 2011). The classical biotype was associated with the previous six pandemics experienced between 1817 and 1923. These pandemics spread from the Indian subcontinent to other continents (Tappero and Tauxe, 2011). In 1935, EI Tor biotype was found to be the major cause of cholera outbreak in Indonesia. It caused a major pandemic in Asia in 1961 spreading to a number of African countries including Zambia, Zimbabwe, Tanzania, and Uganda, slowly replacing the classical biotype (Banerjee et al., 2014). The two biotypes are further divided into Ogawa, Inaba, and Hikojima serotypes based on antigenic factors (Raychoudhuri et al., 2008). Since 1971 when the first outbreak was reported in Kenya, 16 different cholera outbreaks have been reported up to the year 2015 (Tauxe et al., 1995; Shapiro et al., 1999; Mugoya et al., 2008; Scrascia et al., 2009; Shikanga et al., 2009; Kigen et al., 2020). Its recurrence in 2022 clearly indicates that this disease is a major public health threat in Kenya. According to Kenya’s Ministry of Health, the recent 2022–2023 cholera outbreak affected 27 counties (57%) resulting in 12,123 reported cases and 202 fatalities (IFRC, 2024). The ongoing seventh cholera pandemic El Tor strain (7PET) began in Southeast Asia and so far, three transmission waves (I, II, and III) have been identified globally by phylogenetic analysis of which wave III has the largest number of clusters (Weill et al., 2017).

The ability to express virulence factors is essential for *V. cholerae* O1 or O139 to cause epidemics. Several genes have been proposed as virulence markers based on *in vivo* and *in vitro* studies with cholera toxin and toxin coregulated pilus identified as key pathogenic determinants of *V. cholerae* (Faruque et al., 2003). Cholera toxin (*Ctx)* encoded by *ctxA* and *ctxB* genes and carried on the CTXφ prophage is the cause of the severe watery diarrhea seen in cholera patients (Davis and Waldor, 2003). The toxin-coregulated pilus (*tcp*) is responsible for synthesis of fimbriae important for adherence of *V. cholerae* to the intestinal epithelium of the host (Karaolis et al., 1998). In rare cases, toxin coregulated pili (*tcpA*) and cholera toxin (*ctxA*) have been reported in NOVC strains (Singh et al., 2001). Other virulence genes encoding Zonula occludens toxin (*Zot*), accessory cholera enterotoxin (*Ace*), hemolysin (*hlyA)*, repeats-in-toxin A toxin (*rtxA*), and mannose-sensitive hemagglutinin pilus (*mshA*) have been associated with the endemic disease (Feng et al., 2004; Abana et al., 2019).

Mild to moderate cases of cholera are primarily treated by oral or intravenous hydration (WHO, 2024). Antibiotics such as doxycycline and ciprofloxacin are recommended in patients with severe dehydration and those with underlying conditions. Studies have shown that use of antibiotics in this group of patients decreases the duration of diarrhea, volume of stool and the length of shedding of *V. cholerae* (Leibovici-Weissman et al., 2014). A major concern that is linked to this is the emergence of antimicrobial resistant (AMR) strains. Furthermore, the acquisition of mobile genetic elements (MGEs) such as plasmids, transposons, integrons, and integrative conjugative elements (ICEs) plays a significant role in spread of antimicrobial resistance genes (Lassalle et al., 2023; Bhandari et al., 2021).

The pathogenesis and virulence patterns of cholera have been studied both globally and regionally (Chaguza et al., 2024; Kimani et al., 2014; Kiiru et al., 2013; Feng et al., 2004; Abana et al., 2019). Even though surveillance of cholera outbreaks has been done in Kenya, the evolutionary trend and mechanisms driving the emergence and spread of virulent AMR strains remain poorly understood. This study aimed at comprehensively analyzing the genomic data of clinical and environmental *V. cholerae* isolates from the recent 2022–2023 cholera outbreak in Kenya. We performed whole genome sequencing for the isolates in this outbreak and compared them with historical genomes from Kenya, Uganda, Tanzania, and Haiti. We generated data that provides important insights into predicting disease transmission patterns, especially between bordering countries, monitoring the evolution of new variants, and identifying the emergence of virulent and AMR strains. These findings are expected to inform public health strategies for preventing, controlling, and eventually eliminating cholera in Kenya.

## 2 Materials and methods

### 2.1 Sample collection

**FIGURE 1:**
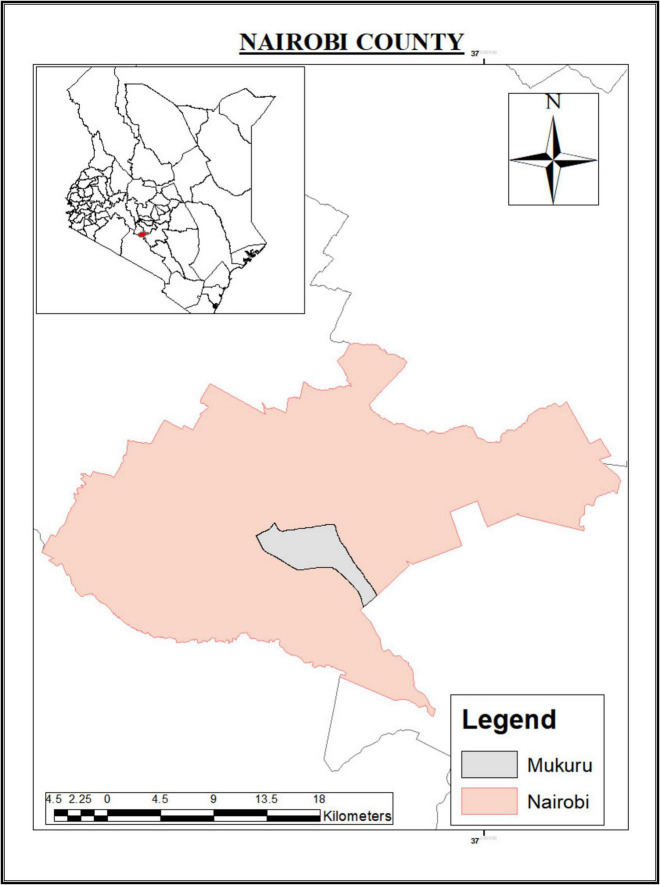
Map showing location of Mukuru informal settlement in Nairobi county.

Environmental samples (wastewater, drinking water, and household effluent) were collected from Mukuru informal settlement in Nairobi county (Figure 1). Clinical samples (stool) were collected from Mukuru and other regions in Nairobi county including Kayole, Mathare, Eastleigh, and Dandora. Mukuru informal settlement, the main sampling site has a population of 770,467 people with 97,890 households according to the 2019 national census report. One percent of residents in Mukuru have access to a private toilet and individual water source (Muriithi and Obare, 2017). Poor hygiene, limited access to clean water, inadequate toilets and poor waste disposal system increase the risk for diarrheal diseases including cholera outbreaks in the study area.

### 2.2 Sampling procedure

#### 2.2.1 Environmental samples

A total of 121 environmental samples were collected during the epidemic period of January to March 2023 from randomly identified households in the study area. These included 40 drinking water samples, 41 wastewater samples and 40 household effluent samples. Drinking water samples were collected in sterile 100 ml Whirl-Pak bags with or without sodium thiosulfate tablets, following determination of chlorine concentration. Chlorinated water was collected into Whirl-Pak bags with sodium thiosulfate tablets. Household effluents, which majorly included gray water from kitchens, laundry facilities, and baths, were collected using a sterile ladle. Upon ensuring the samples were free of sediments, we carefully transferred the samples into 2-L sterile labeled Whirl-Pak bags. Wastewater containing human waste and household effluent was collected from open drains using the same method as described above for household effluent sampling. All samples in the Whirl-Pak bags were transported to the Centre for Microbiology Research Laboratory at Kenya Medical Research Institute (KEMRI) in cool boxes packed with ice for processing within 6 h of collection.

#### 2.2.2 Clinical samples

Stool samples were collected in clean disposable polypots from suspected cholera patients (patients aged 2 years or more with acute watery diarrhea with or without vomiting) attending hospitals in the study areas in November and December 2022. This was based on Integrated Disease Surveillance and Response Standard case definitions.^1^ Diarrheal stool samples were transported to the Centre for Microbiology Research Laboratory at KEMRI using Cary Blair transport media (Oxoid, Thermo Fisher Scientific, USA) within 2 h of collection.

### 2.3 Laboratory analysis

#### 2.3.1 Environmental samples

We measured the Turbidity and PH of each of the samples upon receipt in the laboratory in order to assess sample quality and identify any physicochemical characteristics that could affect *V. cholerae* recovery. For drinking water, 10 ml of each sample was filtered through a membrane filter of 0.45 μm pore diameter (Millipore, Bedford, MA, United States). The filter paper was transferred into 10 ml Alkaline Peptone Water (APW), well shaken and incubated at 37°C for 18 h. Twenty-five (25) ml of each sample of wastewater and household effluent was added to 25 ml of APW in a conical flask and incubated at 37°C for 18 h. Following enrichment, we streaked the samples onto thiosulfate citrate bile salts sucrose agar (TCBS) plates (Oxoid Ltd.) and incubated at 37°C for 18 h. Characteristic small to large yellow colonies were subcultured on Muller Hinton (MH) agar and incubated for 18 h. Purified isolates were then subjected to oxidase test as described previously by Hounmanou et al. (2016). Those that turned positive were presumed to be *V. cholerae* isolates. We extracted DNA from pure cultures of suspected *V. cholerae* colonies using the Zymo Quick-DNA fungal/bacterial Miniprep kit (The Epigenetics Company, CA, United States). Extracted DNA was subjected to Real Time PCR for detection of *V. cholerae* species-specific gene *hlyA* based on a highly specific protocol by Huang et al. (2009). Briefly, the 25 μl master-mix comprised 0.4 μM of each *hlyA* forward and reverse primers, 12.5 μl Bio-Rad iQ Multiplex Powermix, 5 μl molecular water, 0.2 μM *hlyA* probe, and 5 μl of DNA template. AMPLIRUN *V. cholerae* DNA control (VIRCELL Microbiologists, MBC118) served as the positive control while nuclease-free water was used as the negative control. Quantitative PCR was run on a Magnetic Induction Cycler (MIC) using the following conditions: activation (at 95°C for 15 min) and 40 cycles of 95°C for 15 s, 55°C for 40 s, and 72°C for 30 s. PCR reactions were duplicated for each sample. We classified cultures with Ct values of 20 or lower and a difference of less than 2 between duplicate Ct values as positive.

#### 2.3.2 Clinical samples

Stool samples were streaked onto TCBS agar plates (Oxoid Ltd.) following enrichment in APW and incubated at 37°C for 18 h. Characteristic small to large yellow colonies were subcultured on MH agar and incubated for 18 h. Purified isolates were then subjected to oxidase test as described by Hounmanou et al. (2016). Those that turned positive were presumed to be *V. cholerae* isolates. In order to confirm serotype identity, serology was carried out using polyvalent, anti-Ogawa, and anti-Inaba antisera (Denka Seiken, Tokyo, Japan). A similar protocol for DNA extraction and Real Time PCR as for environmental samples was employed for clinical sample analysis.

A total of 100 μl DNA aliquots for 137 positive cultures of clinical (*n* = 120) and environmental (*n* = 17) isolates were shipped on dry ice to Ohio State University for library preparations and whole genome sequencing. We stored all confirmed positive isolates at −80°C for further analysis.

### 2.4 Phenotypic antimicrobial susceptibility testing for clinical and environmental samples

We screened 70 clinical and 17 environmental isolates for antimicrobial susceptibility using the Kirby-Bauer disk diffusion method (Biemer, 1973). The 70 clinical samples screened for AST were randomly selected from the 120 positive isolates with each of the sampling sites represented. The following antimicrobial agents were used; ampicillin (10 μg), gentamicin (30 μg), ciprofloxacin (5 μg), nalidixic acid (30 μg), ceftazidime (30 μg), trimethoprim sulfamethoxazole (25 μg), ceftriaxone (30 μg), cefpodoxime (30 μg), tetracycline (30 μg), azithromycin (15 μg), amoxicillin-clavulanate acid (30 μg), chloramphenicol (30 μg), cefotaxime (30 μg), and Kanamycin (25 μg). Potency of the antibiotic discs and growth of bacteria was tested using *Escherichia coli* ATCC 25922 as the control.

Briefly, *V. cholerae* isolates were grown on MH agar plates for 24 h at 37°C. Colonies were emulsified into normal saline to achieve a 0.5 MacFarland suspension. This suspension was evenly spread onto MH agar (Oxoid, Basingstoke, UK) and allowed to air dry. After air drying the antibiotic disks were applied on the agar and incubated at 37°C for 24 h. We measured the diameter of the inhibition ring and determined susceptibility based on 2018 Clinical and Laboratory Standards Institute (CLSI) M45 established guideline for infrequently isolated or fastidious bacteria (CLSI, 2018). We classified isolates showing resistance to at least three categories of antibiotics as multidrug resistant (MDR). Whole genome sequencing analysis was used to detect AMR genes.

### 2.5 Whole genome sequencing

Genomic DNA of 137 isolates [environmental (*n* = 17) and clinical (*n* = 120)] were subjected to whole genome sequencing. We included one clinical isolate from the 2016 cholera outbreak. Library preparation was done using an Illumina paired-end DNA library preparation kit. Briefly, the 4150 Tapestation system was used to determine library size and concentration (Agilent, MA, USA). In order to amplify the tagged DNA and introduce sequencing indexes, the limited-cycle PCR was subsequently employed. We incorporated PhiX Control v3 (Illumina, Inc., San Diego, CA, USA) into each sample prior to library preparation to facilitate a limit of detection assessment for each sample. The prepared libraries were loaded onto a reagent cartridge and subjected to clustering on the NextSeq 2000 System. Subsequently, a paired-end sequencing run with 2 × 150 bp reads was executed using the NextSeq 2000 platform. Raw sequences have been deposited in the National Centre for Biotechnology Information (NCBI) Sequence Read Archive (SRA) BioProject number PRJNA1235657. The SRA accession numbers and other metadata for each sample are provided in Supplementary Table 1.

### 2.6 Genomic analysis

#### 2.6.1 Serotyping, biotyping, identification of major virulence genes and sequence types, characterization of pathogenicity islands, and assessment of phage susceptibility

Assembly of the raw paired-end reads was done using SPAdes assembler (Bankevich et al., 2012). The assembled sequences were analyzed using CholeraeFinder 1.0 tool in the Centre for Genomic Epidemiology (CGE) platform.^2^ We confirmed cholera species based on the species-specific o*mpW* gene (Siriphap et al., 2017) with a threshold set at 98% identity. This tool further identified *V. cholerae* serogroup-specific genes (*rfbV*-O1 and *wbfZ*-O139), biotype-specific genes (*ctxB, rstR*, and *tcpA*), the gene specific for the 7th pandemic (*VC2346*), and putative virulence genes. Detection of Vibrio pathogenicity islands mainly VPI-1, VPI-2, VSP-1, VSP-2, and PICI like elements responsible for phage susceptibility (PLE1 and PLE2) was also carried out using CholeraeFinder 1.0. Multilocus sequence typing was done using the bactopia bacterial analysis pipeline available from https://bactopia.github.io/latest/ for short paired-end reads. In the bactopia pipeline, contigs were subjected to the “MLST_MODULE” to determine the sequence type. Briefly, the contigs were queried against a custom Multilocus Sequence Typing (MLST) database (build 2.23.0-20240325) using MLST (version 2.23.0) with automatic detection of scheme, 100% identity, minimum depth of sequence coverage of 10 and minimum alignment score of 50. Additionally, the assembled contigs were annotated using prokka version 1.14.6.

#### 2.6.2 Identification of antimicrobial resistance genes and mobile genetic elements

Antimicrobial resistance genes in the assembled contigs were detected using amrfinderplus (version 3.12.8) against the amrfinderplus-database (build 2024-01-31.1) in the bactopia pipeline. Additional search for MGEs, the different classes of integrons and mutations in *gyrA* and *parC* genes (Siriphap et al., 2017) was done by CholeraeFinder on the CGE platform. The PlasmidFinder 1.3 tool of the bactopia pipeline in CGE was used to search for plasmid replicons.

#### 2.6.3 Single nucleotide polymorphism-based phylogenetic analysis

In order to identify the phylogenetic relatedness within and between clinical and environmental *V. cholerae* isolates, high quality assembled genomes were mapped against a reference genome strain of *V. cholerae* N16961 (GenBank accession numbers NZ_LT906614 and NZ_LT906615) using Snippy (version 4.6.0).^3^ Single nucleotide polymorphisms (SNPs) were called with Freebayes (version 1.3.2) using these parameters: minimum mapping quality 60, minimum base quality 13, minimum read coverage 4, and minimum proportion of variant evidence of 75%. With the called SNPs, a core-SNP alignment was then generated. A distance matrix of the SNPs was computed using snp-dists (version 0.8.2) and recombination masked with Gubbins version 3.3.1 (Croucher et al., 2015). A maximum likelihood phylogenetic tree was constructed using IQTREE version 2.2.2.7 (Nguyen et al., 2015) with 1000 ultrafast bootstrap replicates under the HKY model (Posada and Crandall, 2001) and the final tree amended in iTOL.

#### 2.6.4 Global phylogenetic analysis

To understand the evolutionary and temporal dynamics of *V. cholerae* beyond the 2022–2023 sequenced genomes, we retrieved genomic data of *V. cholerae* isolated in different countries from pathogen watch.^4^ The dataset of complete genomes of *V. cholerae* used in evolutionary and temporal dynamics comprised 606 isolates (study isolates: *n* = 105; pathogenwatch isolates: *n* = 501). The 501 included sequences from Kenya (*n* = 106), Tanzania (*n* = 69), Uganda (*n* = 17), and Haiti (*n* = 308), with the M66 strain used as an outgroup. M66 strain genome was the earliest and ancestral sequence among the seventh pandemic isolates, collected in Indonesia in 1937. We applied the bactopia main module to generate assemblies followed by variant calling with Snippy as described above with inclusion of high-quality genome assemblies. The masked core SNP alignment (*n* = 322) with 60800 SNPs was used for phylogenetic analysis with M66 strain as an outgroup.

To understand the temporal dynamics of *V. cholerae* in Kenya, we randomly grouped the genomes by collection time (year), then selected at least 20 genomes per year yielding 37 sequences spanning from 1985 to 2022. For the “global” dataset comprising sequences from the other countries (Kenya, Tanzania, Uganda, and Haiti), we subsampled the genomes by year and selected 12 genomes per sampling time, yielding 94 isolates. For each dataset, maximum phylogenetic inference was performed using IQTREE with generalized time reversible (GTR) applied as the optimal substitution model for evolution (Substitution Models, 2024). To perform phylodynamic analysis, we assessed the temporal signal of the subsampled genome sequences by regressing the genetic distances against collection time (years). Having ascertained sufficient temporal signal, we performed phylodynamic analysis incorporating the sampling locations as traits for phylogeographic reconstructions for dispersal dynamics. For the genomes generated in this study, we inferred the longitude and latitude coordinates using “tidygeocoder” in R using the sampling sites as locations. The associated metadata of the pathogenwatch retrieved genomes were bundled with longitude and latitude information as other relevant annotations. We used Bayesian evolutionary analysis sampling trees (BEAST) version 1.10.4 (Suchard et al., 2018) to perform phylodynamic analysis. For both subsampled datasets, we applied the sampling date (in years) as the tip dates, GTR as the best evolution and Skyline demographic/population models. We used continuous phylogeographic inference by adding the location traits given as longitude and latitude geographical coordinates under a Cauchy distribution model. For the subsampled isolates from Kenya, we run BEAST for 100 million chains, sampling every 10,000th step while the global dataset was run for 300 million generations with sampling at every 30,000th step. We assessed the mixing properties of the relevant estimates for convergence, ensuring effective sample sizes (ESS) > 200 was attained using Tracer version 1.7.2 (Rambaut et al., 2018). Maximum clade credibility trees were obtained using Tree Annotator version 1.10.4 (Rambaut et al., 2018) with a 10% burn-in. Estimates of parameters of interest were reported as median values at 95% highest posterior density or credible interval.

The Phylogenetic tree was constructed using IQ-Tree version 2.0.3 with M66 used as an outgroup and the final tree amended in iTOL (Letunic and Bork, 2021). Accession numbers of strains used in the SNP tree are reported in Supplementary Table 2.

### 2.7 Ethics statement

Ethical approval for this study was obtained from the Kenyatta National Hospital-University of Nairobi Ethical Review Committee (KNH-UON ERC). The Institutional Review Board reviewed the procedures outlined in this study to ensure the protection of human subjects, the privacy of participants, and the ethical conduct of research (Approval ID: P731/09/2021). Research license was sought from the National Commission for Science, Technology, and Innovation (License No.: NACOSTI/P/22/16171).

## 3 Results

### 3.1 Genomic characterization and virulence profiles of clinical and environmental *Vibrio cholerae* isolates

Out of the 137 sequenced isolates, 105 genomes were included in the final genomic analysis comprising 95 genomes from clinical samples and 10 from environmental samples. The remaining genomes were excluded due to less than 50% coverage when mapped to the N16961 reference strain, thus failing quality control checks. The assembled genomes of Kenyan isolates included in the final analysis are available publicly at the NCBI GenBank (BioProject number: PRJNA1235657), with accession numbers and metadata of each sample provided in Supplementary Tables 3, 4 for clinical and environmental isolates respectively.

Ninety-eight percent (*n* = 93) of the sequenced strains from the 2016 (*n* = 1) and 2022 (*n* = 92) clinical isolates were characterized as serogroup 01, containing both *ompW* and *rfbv*-O1 genes. The remaining two isolates were identified as non O1/non-O139. All clinical isolates belonged to the third wave and carried the genetic marker for 7th pandemic *V. cholerae* strains (*VC2346* gene). These isolates were of Ogawa serotype and carried *ctxB7* genotype of the ctxB gene. Additionally, *V. cholerae* O1 isolates contained the *rstR* and *tcpA* genes found in El Tor biotype. Multi-locus Sequence Typing revealed that all the clinical strains belonged to sequence type 69 (ST69). There was a similar trend in occurrence of virulence-associated genes and pathogenicity islands across all clinical strains except one isolate that lacked the *ctxB* gene. This included the key virulence genes such as *ctxA, ctxB, zot, ace, hlyA, mshA, als, makA, rtxA, ompU*, and *toxR.* The *chxA and stn* genes were absent in all strains. Additionally, Vibrio pathogenicity islands VPI-1, VPI-2, VSP-1, and VSP-2 were detected in all clinical strains as shown in Table 1 and Supplementary Table 3.

**TABLE 1:** Genomic characterization of clinical and environmental isolates from the 2022 to 2023 cholera outbreak in Kenya.

Attribute	Clinical Isolates	Environmental isolates
Species	*Vibrio cholerae*	*Vibrio cholerae*
Serogroup/biotype	O1, El Tor, Non O1-non-O139	Non O1-non-O139
ST or clone	ST69	ST 596, ST1272, ST1443, novel STs
Genomic wave 7th pandemic gene (VC2346)	Wave III Present	Not detected Not detected
Pathogenicity islands	VSP-1, VSP-2, VPI-1, VPI-2	VPI-2, VSP-2

All the 10 environmental isolates neither harbored *rfbv*-01 nor *wbfZ*-0139 genes hence were classified as non-O1 and non-O139 *V. cholerae* (NOVC). Genomes of some of the isolates had virulence factors such as *Vibrio* pathogenicity islands VPI-2 (*n* = 1) and VSP-2 (*n* = 1). Additionally, *toxR, als*, and *hlyA* genes were found in all environmental isolates. The *rtxA* gene was found in 90% of these isolates. *In silico* MLST revealed that three strains belonged to sequence types 596, 1272, and 1443 with majority (70%) belonging to Novel STs. Both clinical and environmental strains harbored *toxR*, *als*, *hlyA*, and *rtxA* genes. This is shown in Table 1 and Supplementary Tables 3, 4.

### 3.2 Phenotypic antibiotic resistance profiles in clinical and environmental *Vibrio cholerae* isolates

Both clinical and environmental isolates phenotypically expressed antibiotic resistance. The seventy randomly selected clinical isolates had an identical multidrug resistance profile, showing 100% resistance to ampicillin, cefotaxime, ceftriaxone, cefpodoxime, trimethoprim sulfamethoxazole, nalidixic acid, and azithromycin. Susceptibility to gentamicin and chloramphenicol was observed in all clinical strains. The clinical isolates were highly susceptible to tetracycline (99%) while 96% of the isolates exhibited intermediate susceptibility to ciprofloxacin (Table 2). All environmental isolates were susceptible to azithromycin and gentamicin. Phenotypic resistance to ampicillin, trimethoprim sulfamethoxazole, and nalidixic acid was observed in 59%, 41%, and 47% of the environmental isolates respectively as shown in Table 2.

**TABLE 2:** Phenotypic antibiotic resistance profile of clinical and environmental isolates.

Antibiotic	Clinical isolates			Environmental isolates		
	Susceptible *n* (%)	Intermediate *n* (%)	Resistant *n* (%)	Susceptible *n* (%)	Intermediate *n* (%)	Resistant *n* (%)
Ampicillin	0 (0)	0 (0)	70 (100)	6 (35.3)	1 (5.9)	10 (58.9)
Ceftazidime	0 (0)	1 (1.4)	69 (98.6)	15 (88.2)	0 (0)	2 (11.8)
Cefotaxime	0 (0)	0 (0)	70 (100)	14 (82.4)	1 (5.9)	2 (11.8)
Amoxicillin clavulanate	36 (51.4)	32 (45.7)	2 (2.9)	13 (76.5)	3 (17.6)	1 (5.9)
Ciprofloxacin	0 (0)	67 (95.7)	3 (4.3)	3 (17.6)	11 (64.7)	3 (17.6)
Azithromycin	0 (0)	0 (0)	70 (100)	17 (100)	0 (0)	0 (0)
Trimethoprim sulfamethoxazole	0 (0)	0 (0)	70 (100)	8 (47.1)	2 (11.8)	7 (41.2)
Chloramphenicol	70 (100)	0 (0)	0 (0)	16 (94.1)	1 (5.9)	0 (0)
Ceftriaxone	0 (0)	0 (0)	70 (100)	14 (82.4)	2 (11.8)	1 (5.9)
Cefpodoxime	0 (0)	0 (0)	70 (100)	13 (76.4)	0 (0)	4 (23.5)
Nalidixic acid	0 (0)	0 (0)	70 (100)	8 (47.1)	1 (5.9)	8 (47.1)
Gentamicin	70 (100)	0 (0)	0 (0)	17 (100)	0 (0)	0 (0)
Kanamycin	60 (85.7)	10 (14.3)	0 (0)	15 (88.2)	2 (11.8)	0 (0)
Tetracycline	69 (98.6)	1 (1.4)	0 (0)	16 (94.1)	0 (0)	1 (5.9)

### 3.3 Antimicrobial resistance genes and mobile genetic elements in clinical isolates

**FIGURE 2:**
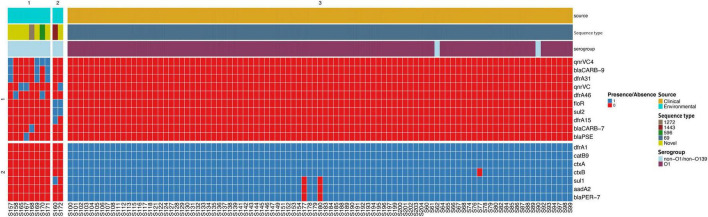
Heat map showing antibiotic resistance genes in clinical and environmental isolates from the 2022 to 2023 cholera outbreak in Kenya. One clinical isolate was collected in 2016. Gene presence is denoted by color blue while red denotes gene absence. The color coded key on the right shows the source of isolates, sequence type, and serogroup.

The *dfrA1* gene, conferring resistance to trimethoprim, was detected in all clinical isolates, consistent with their phenotypic resistance to this antibiotic. Furthermore, the sulfonamide resistance gene *sul1* was identified in 98% of the isolates. Majority (98%) of the isolates harbored the plasmid-borne extended-spectrum beta-lactamase *blaPER-7* gene responsible for third generation cephalosporin resistance. This gene was lacking in the 2016 isolate. Even though all isolates carried the chloramphenicol acetyltransferase gene (*catB9)*, responsible for phenicol resistance, phenotypic resistance to chloramphenicol was not observed. Class 1 integrons, identified by the presence of the *intI* gene, were detected in 99% isolates. The 2016 isolate lacked *intI* gene. However, the SXT integron-related resistance determinants *dfrA18* and the fluoroquinolone resistance gene *qnrVC1* were absent in all samples. Similarly, the SXT-like ICE-borne chloramphenicol resistance gene *floR* and tetracycline resistance genes were not detected, corresponding to the observed 100% and 98% susceptibility of the clinical isolates to chloramphenicol and tetracycline, respectively (Figure 2 and Supplementary Table 3). Ninety-four (99%) isolates carried the plasmid *pVC211*, which is associated with high-level resistance to azithromycin, corresponding to the 100% resistance to this antibiotic. The 2016 isolate lacked *pVC211.* Mutations in *gyrA* and *parC* gene were found in all clinical isolates. Additionally, an IncA/C2 plasmid known to carry multiple antimicrobial resistance genes was found in all clinical isolates as shown in Supplementary Table 3.

### 3.4 Genotypic antibiotic resistance, phage resistance, and mobile genetic elements in environmental isolates

Environmental isolates showed varied resistance profiles. The SXT-like ICE-borne chloramphenicol (CHL) resistance gene *floR* was detected in 20% of the strains. Two isolates carried the sulfonamide resistance gene *sul2* conferring resistance to sulfamethoxazole. The fluoroquinolone resistance gene *qnrVC4* was present in 40% of the isolates. Notably, all isolates lacked *catB9 gene*, a determinant of phenicol resistance. Three isolates carried *bla*_*CARB*–9_ gene, encoding a beta-lactamase enzyme and conferring beta-lactam resistance (Figure 2 and Supplementary Table 4). None of the environmental strains contained the phage susceptibility regions associated with PICI-like elements (PLE1 and PLE2).

### 3.5 Genetic diversity of environmental and clinical *Vibrio cholerae* isolates

**FIGURE 3:**
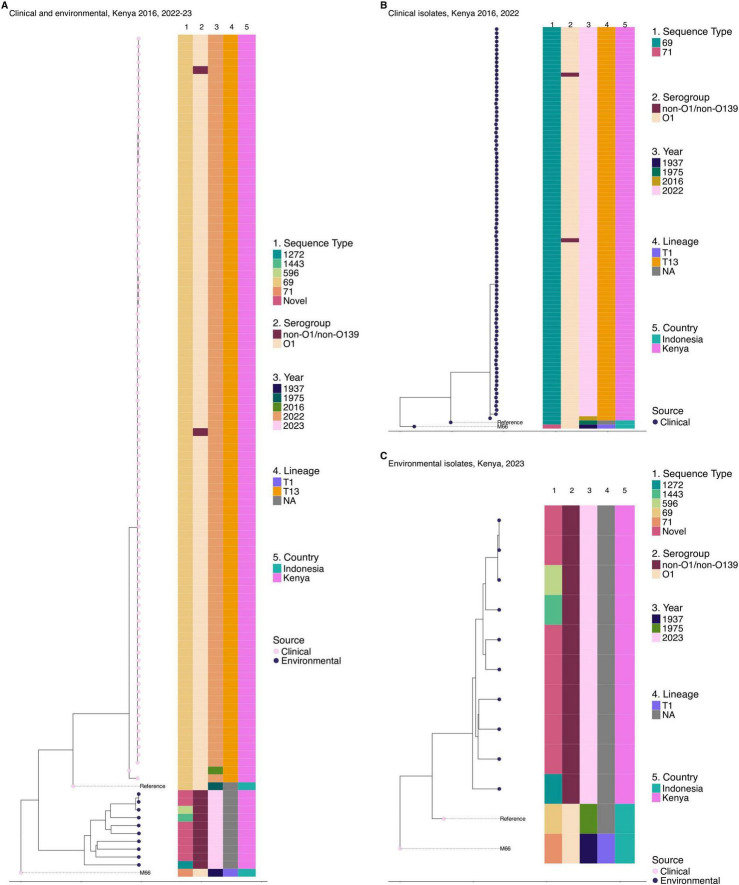
Genetic relatedness of clinical and environmental *V. cholerae* isolates from 2022 to 2023 outbreak in Kenya. One clinical isolate from 2016 was included. **(A)** Maximum likelihood phylogenetic tree showing the genetic relatedness of clinical and environmental isolates collected in the 2022–2023 cholera outbreak in Kenya with one clinical isolate from 2016 outbreak included. **(B)** Maximum likelihood phylogenetic tree illustrating the genetic relatedness of clinical isolates collected in the 2016 (*n* = 1) and 2022–2023 cholera outbreaks in Kenya. **(C)** Maximum likelihood phylogenetic tree illustrating the genetic relatedness of environmental isolates collected during the 2022–2023 cholera outbreak in Kenya. The circles at the tip of the phylogeny represent the sample source. Color strips at the tips of each tree represent the sequence type (ST), year of isolation, serogroup, lineage, and country of origin. NA on the lineage for panels **(A–C)** refers to isolates with no transmission lineage assigned. The phylogeny was constructed based on the reference genome strain of *V. cholerae* N16961 and rooted based on the pre-seventh pandemic strain M66 used as an outgroup.

Phylogenetic analysis of isolates collected during the 2022–2023 cholera outbreak revealed that all the 10 environmental isolates were highly divergent, clustering outside the 7th pandemic El Tor lineage. The difference between these isolates and the reference genome N16961 was between 55,000 and 119,000 SNPs. Multi-locus sequence typing (Jolley et al., 2012) demonstrated significant diversity among the environmental strains with 7 out the 10 isolates classified as novel sequence types that were phylogenetically distinct from previously known types. The other three environmental strains belonged to sequence types (STs) 1272, 1443, and 596 as shown in Figure 3C. This highlights the extensive genetic diversity of *V. cholerae* in environmental reservoirs. Phylogenetic analysis of the 95 *V. cholerae* genomes from clinical isolates revealed a high degree of clonality, with fewer than five SNPs differentiating majority of the isolates. However, the 2016 isolate was an exception showing 15 SNP differences. MLST following the methodology described by Jolley et al. (2012) further confirmed the limited genetic diversity, as all the strains were classified under the same rMLST type, ST69 (Figure 3B). It is important to note that all the clinical *V. cholerae* O1 isolates did not cluster with the 10 environmental isolates (Figure 3A). This further confirms that the clinical strains primarily belonged to the monophyletic 7th pandemic lineage while environmental isolates represent genetically distinct populations.

**FIGURE 4:**
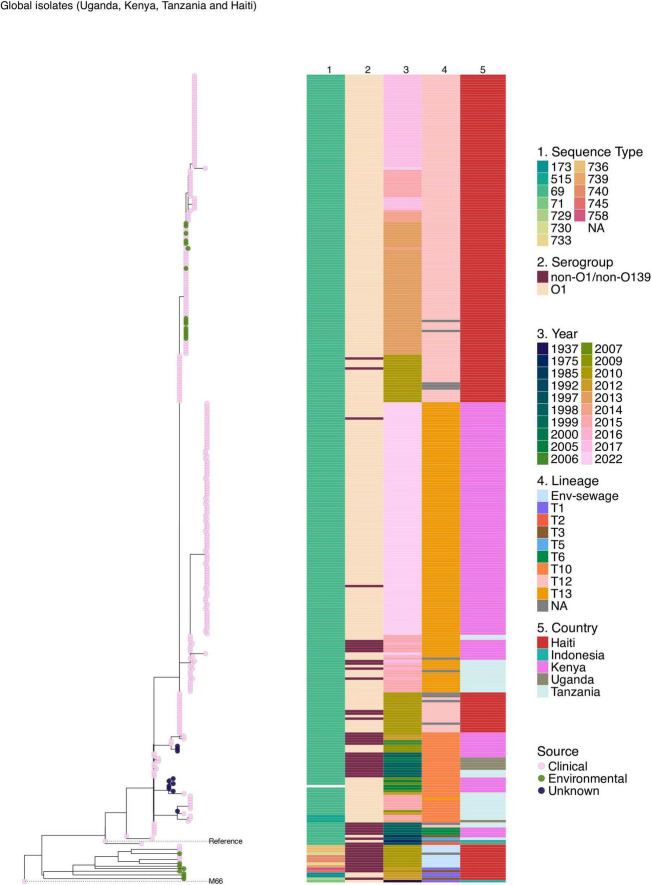
Maximum likelihood phylogenetic tree illustrating the genetic relatedness of clinical and environmental *V. cholerae* isolates collected during the 2022–2023 cholera outbreak in Kenya and previously published genomes from Kenya, Uganda, Tanzania, and Haiti. The pink, green, and blue circles at the tip of the phylogeny represent the sample type. The phylogeny is annotated by color strips at the tips of each tree representing the sequence type (ST), serogroup (SG), year of isolation, lineage, and country of isolation. The phylogeny was constructed based on the reference genome strain of *V. cholerae* N16961 and rooted based on the pre-seventh pandemic strain M66 used as an outgroup.

Given the high burden of cholera in Haiti and Kenya’s bordering countries, Uganda and Tanzania, further analysis was done to place the 2022–2023 Kenyan isolates within the broader phylogenetic framework of East African and North American 7PET lineages. Comparison of clinical genomes from the 2022 to 2023 Kenyan cholera outbreak revealed clonal relatedness to *V. cholerae* O1 El Tor isolates from these regions. The maximum likelihood phylogeny showed that the predominant 2022–2023 outbreak-associated 7PET isolates from Kenya formed a single distinct cluster in the phylogeny and belonged to lineage T13 (Figure 4). The historical 7PET isolates from Kenya clustered with sequences from Tanzania and Uganda. Haiti strains were observed to cluster distinctly with clinical and environmental isolates appearing in the same clusters (Figure 4).

**FIGURE 5:**
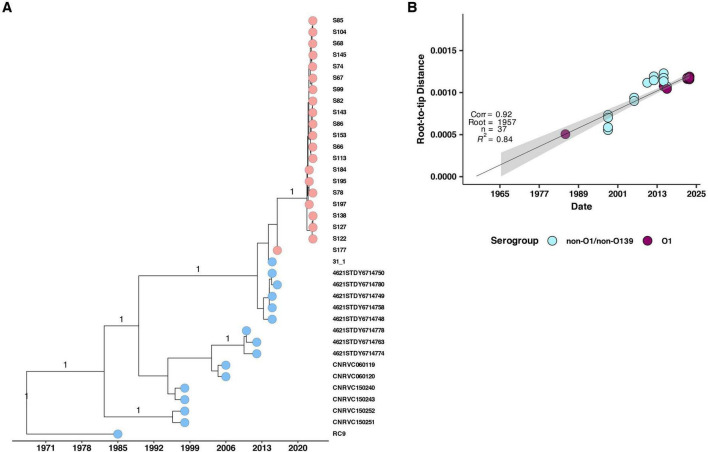
**(A)** Maximum likelihood phylogenetic tree illustrating the genetic diversity of the 2022 Kenyan clinical *V. cholerae* isolates and previously published genomes from Kenya. The pink and blue circles at the tip of the phylogeny represent the isolates with historical isolates up to 2015 denoted by blue while 2016 and 2022 isolates are denoted by pink. The horizontal axis indicates the year of isolation. **(B)** Root to tip distances against collection time (years) on vertical and horizontal axes respectively. We observed a strong correlation (correlation coefficient = 0.92, *R*^2^ = 0.84).

**FIGURE 6:**
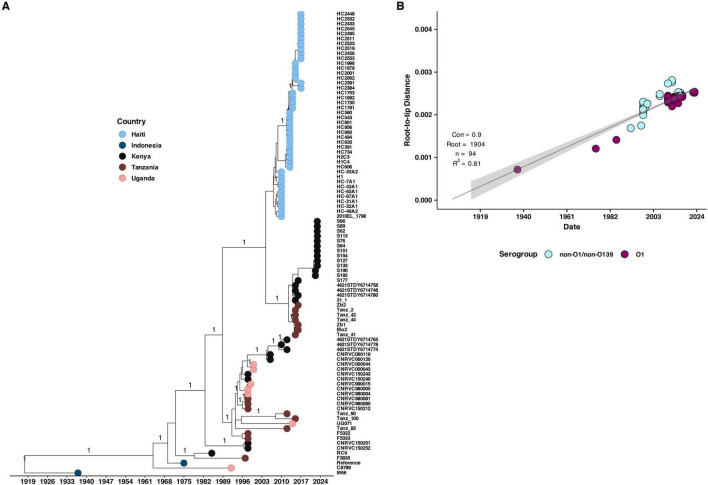
**(A)** Maximum likelihood phylogenetic tree illustrating the genetic diversity of the 2022 Kenyan clinical *V. cholerae* isolates and previously published genomes from Kenya, Uganda, Tanzania, and Haiti. The pink, brown, black, and blue circles at the tip of the phylogeny represent the country of isolation while the horizontal axis indicates the year of isolation. **(B)** Root to tip distances against collection time (years) on vertical and horizontal axes respectively. We observed a strong correlation (correlation coefficient = 0.9, *R*^2^ = 0.81).

To investigate the potential origin of the 2022–2023 cholera outbreak in Kenya, we conducted a time-scaled phylogenetic analysis incorporating previously published Kenyan genomes alongside clinical genomes from the 2022 to 2023 outbreak (Figure 5). Our analysis revealed that the 2022–2023 isolates showed the highest genetic similarity (15 SNPs, lineage T13) to Kenyan isolates from 2016, suggesting a close evolutionary relationship. We further placed the 2022–2023 clinical isolates within a broader phylogeny including genomes from East Africa and North America, which confirmed a similar pattern of genetic relatedness (Figure 6).

## 4 Discussion

Cholera still remains a significant threat to public health since 1971 when the first case was reported in Kenya. Numerous outbreaks have been reported up to the year 2023 highlighting cholera endemic nature in our setting (Tauxe et al., 1995; Shapiro et al., 1999; Mugoya et al., 2008; Scrascia et al., 2009; Shikanga et al., 2009; Mutonga et al., 2013; Kigen et al., 2020). In our study, clinical *V. cholerae* isolates were primarily identified as *s*erogroup O1 El tor variants and carried the key virulence factors responsible for pathogenicity, including the ctxB7 genotype of the *ctxB* gene, as well as the *rstR* and *tcpA* genes. Earlier studies reported ctxB3 toxin allele in all El tor strains in Kenya (Kiiru et al., 2013). In contrast, none of the environmental isolates tested were positive for these genes. This observation agrees with previous studies, which reported the absence of *ctx* and *tcpA* genes in NOVC strains, while all O1 and O139 strains consistently harbored these genes (Faruque et al., 1998; Sharma et al., 1998). However, environmental isolates in our study contained other virulence-associated factors such as VPI-2, VSP-2, and genes including *rtxA*, *als*, and *hlyA*, which have been implicated in diarrheal diseases (Octavia et al., 2013). Among these, the *rtxA* gene is known to encode a product with cytotoxic activity toward mammalian cells, a key factor driving the virulence of CTX-negative, NOVC strains (Lin et al., 1999). Several studies conducted in tropical regions and globally have linked sporadic cases of gastroenteritis to NOVC strains (Sharma et al., 1998; Schwartz et al., 2019; Siriphap et al., 2017). These findings suggest that although environmental isolates lack the virulence cholera toxin genes, they possess alternative virulence genes that may spread among cholera strains via horizontal gene transfer or MGEs and result in gastrointestinal disease. This highlights the need for continued surveillance and characterization of *V. cholerae* circulating in our environment.

In our study, a uniform resistance pattern was observed in all clinical isolates highlighting 100% resistance to multiple antibiotics, including ampicillin, cefotaxime, ceftriaxone, cefpodoxime, trimethoprim-sulfamethoxazole, nalidixic acid, and azithromycin. This resistance pattern contrasts with earlier findings from Kenya which documented lower resistance rates to nalidixic acid (64.5%) and ampicillin (3.6%) (Shah et al., 2023) and high susceptibility to ceftriaxone (99%) (Awuor et al., 2020). Additionally, a study by Haque et al. (2023) reported *V. cholerae* isolates with lower resistance to ceftazidime (27.5%) and cefotaxime (29.4%), findings consistent with reports from other countries (Sahilah et al., 2014; Letchumanan et al., 2015). The mutations observed in *gyrA* and *parC* in clinical isolates indicate quinolone resistance, an important evolutionary trait for sub lineages in the 7th cholera pandemic (Opintan et al., 2021; Weill et al., 2017). Majority of the clinical isolates in our study harbored the plasmid-borne extended-spectrum beta-lactamase *blaPER-7* gene responsible for third generation cephalosporin resistance. This correlated with observed 100% phenotypic resistance to cefotaxime, ceftriaxone, and cefpodoxime. A study done in Yemen reported similar findings with *blaPER-7* gene isolated from multidrug-resistant *V. cholerae* strains (Lassalle et al., 2023). Clinical strains were all susceptible to chloramphenicol despite presence of *catB9* gene possibly due to low gene expression. Similar findings have been reported in a number of studies (Siriphap et al., 2017; Sun et al., 2023) indicating that phenotypic expression is not necessarily related to the presence of the encoding gene. The identification of class 1 integrons in clinical isolates from our study emphasizes their critical role in the acquisition and spread of resistance genes. Notably, these integrons were absent in the sequenced 2016 isolate and in earlier Kenyan studies (Shah et al., 2023) suggesting recent horizontal gene transfer events. The IncA/C2 plasmid found in all clinical isolates in our study is important in horizontal transfer of multiple antimicrobial resistance genes. A previous study in Haiti reported that this plasmid encodes a unique set of resistance determinants and a second copy of the resistance genes hence conferring resistance to multiple antibiotics (Folster et al., 2014). The high number of reported MDR *V. cholerae* clinical isolates in our study could be attributed to this.

These observations suggest an escalating antibiotic resistance scenario over time. Despite this alarming resistance trend, all clinical strains in our study remained susceptible to gentamicin and chloramphenicol while 99% of the isolates were susceptible to tetracyclines. Tetracyclines (doxycycline) have historically been the drug of choice during cholera outbreaks (Poulin-Laprade et al., 2015). It is critical to monitor usage of these antibiotics to reduce the risk of developing resistance as there are fewer therapeutic options now available.

Environmental isolates showed a more varied resistance profile, with 100% susceptibility to gentamicin and azithromycin. Interestingly, three environmental NOVC isolates carried *bla*_CARB–9_ gene conferring beta-lactam resistance. This gene possibly acquired through MGEs has been widely reported in bacteria (De, 2021) with recent studies in Austria and Argentina reporting *bla*_CARB–9_ in environmental NOVC strains (Petroni et al., 2004; Lepuschitz et al., 2019). Environmental isolates also carried unique resistance determinants, including the SXT-like ICE-borne *floR* and *qnrVC4* genes, which confer resistance to chloramphenicol and fluoroquinolones, respectively. Furthermore, the lack of phage susceptibility regions associated with PICI-like elements in environmental strains impacts their persistence in the environment. Previously, bacteriophages infective to *V. cholerae* have been isolated from environmental waters (Maina et al., 2014). There is need to monitor both clinical and environmental strains in order to track the spread of MDR determinants and understand the role of environmental *V. cholerae* in driving emergence of new MDR strains.

The AMR genes circulating in environmental isolates were different from those found in clinical isolates. For instance, the *floR* and *bla*_CARB–9_ genes were detected in environmental isolates but absent in all clinical samples, whereas the *catB9* gene was present in all clinical isolates but absent in environmental isolates. This indicates that the AMR gene profiles in clinical isolates may be evolving differently from those in environmental populations due to a difference in selective pressure.

While majority of clinical isolates clustered together and showed a high degree of genetic relatedness, environmental NOVC isolates were highly divergent with isolates belonging to novel and distinct STs, including ST1272, ST1443, and ST596. This high level of diversity is consistent with previous studies that identified mutation and genetic recombination as key factors driving variation among *V. cholerae* isolates (Octavia et al., 2013; Salim et al., 2005; Feng et al., 2008). Although these environmental isolates clustered outside the 7th pandemic El Tor lineage, they have the potential to cause mild diarrhea and contribute to spread of AMR determinants hence the need for ongoing surveillance to understand the role of environmental isolates in evolution of clinical *V. cholerae* strains.

Comparison of clinical genomes from the 2022 to 2023 Kenyan cholera outbreak with previously published genomes from Kenya, Uganda, Tanzania, and Haiti revealed clonal relatedness to *V. cholerae* O1 El Tor isolates from these regions. This agrees with previous studies (Kiiru et al., 2013; Morita et al., 2020) which showed that *V. cholerae* O1 El Tor isolates in Kenya and countries in Southeast Asia are clonally related to strains from other regions globally. Similarly, Stine and Morris (2013) reported that isolates from the sixth and seventh pandemics share a single ancestral origin. The historical 7PET isolates from Kenya clustered with sequences from Tanzania and Uganda suggesting cross border spread of cholera. This was in agreement with an earlier study that reported cross border cholera outbreaks as one of the major contributors to the high cholera burden in Sub Saharan Africa (Bwire et al., 2016). Although there is no direct evidence of recent transmission events between Haiti and Kenya, the shared ancestral lineage of isolates from the sixth and seventh pandemics highlights the potential for global dissemination of epidemic *V. cholerae* strains through international travel and trade. The cholera epidemic that affected Haiti from October 2010 to February 2019 has been attributed to the introduction of *V. cholerae* by United Nations peacekeepers originating from South Asia (Piarroux et al., 2011). This shows the role of human movement in the transcontinental spread of cholera further highlighting the importance of regional and global surveillance in effectively monitoring and controlling the spread of cholera. Clinical and environmental isolates from Haiti appeared in the same clusters suggesting environmental persistence and possible spill over to human populations.

The 2022–2023 cholera outbreak isolates showed the highest genetic similarity (15 SNPs) to Kenyan isolates from 2016, suggesting a close evolutionary relationship. These results indicate that the 2022–2023 outbreak did not arise from a new introduction but instead resulted from re-emergence of previously circulating strains in Kenya that had persisted since 2016. Sporadic cholera outbreaks were reported in Kenya each year from 2016 to 2022.

While we examined clinical genomes from the 2022 to 2023 cholera outbreak alongside genomes from previous outbreaks in Kenya, Uganda, Tanzania, and Haiti, including more countries would have provided a better global perspective on cholera transmission patterns. However, the selected regions offer important insights into *V. cholerae* genomics within regional and global contexts. Future genomic surveillance studies incorporating more countries will further enhance our understanding of the spread and genetic diversity of *V. cholerae* globally.

## 5 Conclusion

In conclusion, our study shows that the 2022–2023 Kenyan cholera outbreak has been attributed to 7PET O1 ogawa *V. cholerae* strains carrying IncA/C2 plasmids and multidrug resistant genes and likely resulted from re-emergence of previously circulating strains rather than a new introduction. Kenyan clinical isolates remain susceptible to tetracycline, gentamicin, and chloramphenicol. Although environmental contamination as a source of human infection cannot be clearly elucidated in our study, it is important to note that environmental isolates possess virulent and AMR genes that may spread to clinical and other environmental strains via horizontal gene transfer or MGEs. This highlights the need for continued surveillance in order to track *V. cholerae* evolution, understand transmission pattern, and limit the development and spread of antimicrobial resistance.

## Data Availability

The datasets presented in this study can be found in online repositories. The names of the repository/repositories and accession number(s) can be found in the article/Supplementary material.

## Footnotes

1
https://www.nphi.go.ke/sites/default/files/2024-02/IDSR%20Clinicians%20Handbook.pdf

2
https://cge.food.dtu.dk/services/CholeraeFinder/

3
https://github.com/tseemann/snippy

4
https://pathogen.watch/
